# Comparison of culture, ELISA and PCR techniques for salmonella detection in faecal samples for cattle, pig and poultry

**DOI:** 10.1186/1746-6148-3-21

**Published:** 2007-09-22

**Authors:** Erik Eriksson, Anna Aspan

**Affiliations:** 1 National Veterinary Institute, Department of Bacteriology, SE-751 89 Uppsala, Sweden

## Abstract

**Background:**

Performances of different salmonella detection methods were evaluated by applying them to of artificially contaminated faecal specimens from cattle, pigs and poultry. The NMKL71 method, being the standard reference method for detection of salmonella in the official Swedish control program, was compared with the proposed ISO method using MSRV-selective enrichment for culturing, and also with three commercial ELISA- based systems, Bioline Selecta, Bioline Optima and Vidas, a commercial PCR-based method, BAX^® ^system, and three different strategies using PCR detection using a non-commercial PCR system.

**Results:**

Altogether, 391 samples were tested, and the overall results clearly indicate that, when faeces from all animal species and all serotypes were included, the MSRV performed best, with a calculated accuracy of 99% and a calculated sensitivity of 98%. The second most sensitive and specific method was the BAX^® ^system, using the modified enrichment protocol as recommended by the manufacturer for faecal samples. However, this protocol includes one additional day of work, as compared with the standard procedure for food sample analysis by the same method. The different strategies for salmonella detection using non-commercial PCR showed a sensitivity and specificity in the same range as the BAX^® ^method; furthermore, results were obtained more quickly. The various commercial ELISA methods and the NMKL method showed the poorest performance of the methods included in the study, and were closely dependent on the origin of the faeces used and on which salmonella strain was to be detected.

**Conclusion:**

The study showed that the sensitivity of the different methods depended to a great extent on the origin of the faecal matrices and the salmonella strains used to "spike" the samples.

## Background

Salmonella in Swedish food producing animals is rare, due mainly to vigorous salmonella control program (SCP) initiated in the 1960s. The strategy of this program is to monitor the presence of salmonella at all stages in the " farm to fork" production chain, and to intervene whenever salmonella is found. In the Swedish salmonella control at slaughter, the data for salmonella positive samples in 2004 were: 0.04% for cattle, 0.06% for pigs and 0.08% for poultry [1].

In 2003, however, the largest out-break of salmonella in Swedish animal production hitherto was reported [1], a routine pig faecal sample in the control program run by the industry tested positive for S. Cubana. Trace-back investigation revealed that provender purchased by the farm concerned, was contaminated with S. Cubana. A feed factory had supplied provender to several farms, mainly pig producers, but also several cattle farms. Altogether, 49 farms were identified as having either salmonella-positive pigs and/or contaminated feed. However, no human cases of S. Cubana were reported, nor did any samples of food origin prove positive for S. Cubana during that year. In all, only 0.2% of tested food samples were salmonella positive in 2004.

In the investigation following the "Salmonella Cubana outbreak", time was identified as a crucial factor in cost reduction, and rapid methods for the detection of salmonella were urgently sought. For the SCP in Sweden, the stipulated method to be used is NMKL71 [2]. When using this protocol the first indication as to whether a sample is positive or negative is obtained on day 3 (if the analysis is started on day 0). Assumed positive samples must be confirmed, and a final result is determined after 1 to 2 additional days. In addition to the SCP, many Swedish companies perform their own HACCP-based salmonella surveillance, and in such cases, any validated salmonella detection method may be used.

In 2004, the Swedish Board of Agriculture decided that a study should be conducted to evaluate alternative rapid methods for salmonella detection that could be candidates to replace NMKL71 in critical investigations concerning farm animals, especially for faecal samples, where the choice of method may be suspected to appreciably influence the test results. Such methods could be ELISA-based and PCR-based techniques.

A number of procedures were initially considered for inclusion in the study, but for various reasons some were rejected, or were excluded at some stage of the investigation. The procedures presented here are: two culture-based methods, NMKL71 and Draft Amendment ISO 6579:2002/amendedDAmd 1, three commercially available ELISA methods (Vidas SLM, Bioline Optima and Bioline Selecta) and one commercially available PCR method (the BAX^® ^system). In addition, culturing from the enrichment broths of the Bioline Optima and the Bioline Selecta systems was performed, and finally a non-commercial PCR method was used to evaluate the possibility of performing PCR analysis after different stages of pre-enrichment or selective enrichment. Furthermore, for spiked faecal cattle samples an additional culture- based method (Selenite/Cystine enrichment) was evaluated.

The EU's reference laboratory for salmonella (CRL) has organized comprehensive inter-laboratory comparison studies for different salmonella detection methods among the EU's national reference laboratories (NRLs) of member states [3,4]. These studies performed on both artificially and naturally contaminated samples have shown that MSRV are more sensitive than methods based on RVS enrichment (the enrichment used in the NMKL71 method). Both methods include a pre-enrichment in buffered peptone water (BPW), but the second enrichment is done either on modified semisolid Rappaport-Vassiliadis agar (MSRV) or in Rappaport-Vassiliadis sojapepton broth (RVS), with over night incubation.

In a study [5] on faecal samples from pigs (n = 1591), calves (414) and cows (456), MSRV enrichment was found to be significantly more sensitive than RV enrichment when analysing pig samples, whereas no difference could be discerned in cattle samples. The performance of MSRV was significantly better compared to RV enrichment also for poultry faecal samples, according to a study published by Voogt et al. [6]. RV (Rappaport-Vassiliadis magnesium chloride/malachite green medium broth) was the broth used in the 3rd edition of the ISO method (ISO 8579:1993 [E]) for salmonella detection, but an improvement has since been made to this broth, including soy-peptone and also using a different concentration of magnesium chloride.

The Vidas system has been evaluated by Korsak et al, [7] for salmonella analysis of animal faeces. In this study the authors found that Vidas was comparable to the NMKL71 method. It has also been evaluated for faecal samples, with promising results, by Sommerhauser & Failing [8].

Regarding the Bioline methods (Optima and Selecta) included in this study, as far as we know, nothing has been published on analyses of faecal samples.

A few reports have appeared on diagnostic PCR testing using faecal samples but to our knowledge no comprehensive comparison of culture vs. PCR diagnostics. However, there are reports [9,10] indicating that PCR diagnostics using faecal samples can give e results comparable to culture methods.

The aim of the present study was to perform the evaluation as instructed by the Swedish Board of Agriculture, in order to assess potential rapid replacement methods for the NMKL71 method. Furthermore, the MSRV method, suggested to be a new annex to the ISO 6579 standard [11] and which is the method recommended by the EU's CRL for salmonella for analysis of salmonella in faecal samples [12] was included in the present study.

## Results

All our results are summarized in Tables 1, 2, 3, 4, 5, including the statistical analysis data. Table 1 summarizes the results of all analyses performed in study

**Table 1:** Summary of all results. Results of the analyses of the 100 pooled faecal samples originating from 265 assumed salmonella-free farms and 3 control samples combined with the results from trials 1–9, artificially contaminated poultry, swine and cattle faecal samples.

	**NMKL**	**MSRV**	**MSRV PCR**	**VIDAS SLM**	**SELECTA ELISA**	**SELECTA CULTURE**	**OPTIMA ELISA**	**OPTIMA CULTURE**	**BAX^®^**	**SELECTA PCR**	**SELECTA IMS PCR**
TP*	128	226	226	109	145	203	52	114	196	188	181
TN*	154	154	154	154	154	154	154	154	153	154	154
FP*	0	0	0	0	0	0	0	0	1	0	0
FN*	109	5	5	128	85	27	179	117	26	36	43

AC**	0.72	0.99	0.99	0.67	0.78	0.93	0.54	0.7	0.93	0.9	0.89
SE**	0.54	0.98	0.98	0.45	0.63	0.88	0.23	0.49	0.88	0.84	0.81
SP**	1	1	1	1	1	1	1	1	0.99	1	1

*TP = True positives, **TN **= True negatives, **FP **= False positives, **FN **= False negatives

**AC = Accuracy, **SE **= Sensitivity, **SP **= Specificity

**Table 2:** Results from 100 pooled samples consisting of assumes Salmonella-free faeces. Assumed salmonella-free faecal samples originating from 265 farms (poultry, pig, and cattle) were analysed as 100 pooled samples, each consisting of 25 g of faeces; no salmonella bacteria added. In parallel, three control samples, spiked with 300 cfu *Salmonella *Typhimurium DT1, 2800 cfu *Salmonella *Worthington, and 5000 cfu *Salmonella *Typhimurium DT40 respectively, were analysed.

**CFU salm/25 g**	**NMKL**	**MSRV**	**MSRV PCR**	**VIDAS SLM**	**SELECTA ELISA**	**SELECTA CULTURE**	**OPTIMA ELISA**	**OPTIMA CULTURE**	**BAX^®^**	**SELECTA PCR**	**SELECTA IMS PCR**
**0**	0/100	0/100	0/100	0/100	0/100	0/100	0/100	0/100	1/100	0/100	0/100
**Control**	3/3	3/3	3/3	2/3	3/3	3/3	2/3	2/3	3/3	3/3	3/3

TP*	3	3	3	2	3	3	2	2	3	3	3
TN*	100	100	100	100	100	100	100	100	99	100	100
FP*	0	0	0	0	0	0	0	0	1	0	0
FN*	0	0	0	1	0	0	1	1	0	0	0

AC**	1.0	1.0	1.0	0.99	1.0	1.0	0.99	0.99	0.99	1.0	1.0
SE**	1.0	1.0	1.0	0.67	1.0	1.0	0.67	0.67	1.0	1.0	1.0
SP**	1.0	1.0	1.0	1.0	1.0	1.0	1.0	1.0	0.99	1.0	1.0

*TP = True positives, **TN **= True negatives, **FP **= False positives, **FN **= False negatives

**AC = Accuracy, **SE **= Sensitivity, **SP **= Specificity

**Table 3:** Results from trial 1–3; poultry faecal samples artificially contaminated with salmonella. Faeces originating from two egg-producing farms (Trial 1), two egg-producing farms and one broiler farm (Trial 2–3). For each trial different spiking levels of salmonella were constructed (Trial 1 *S*. Enteritidis, Trial 2 *S*. Livingstone, Trial 3 *S*. Worthington), each spiking level containing six 25 g samples of faeces.

**CFU salm/25 g**	**NMKL**	**MSRV**	**MSRV PCR**	**VIDAS SLM**	**SELECTA ELISA**	**SELECTA CULTURE**	**OPTIMA ELISA**	**OPTIMA CULTURE**	**BAX^®^**	**SELECTA PCR**	**SELECTA IMS PCR**
**TRIAL 1**											
**0**	0/6	0/6	0/6	0/6	0/6	0/6	0/6	0/6	0/6	0/6	0/6
**5000**	6/6	6/6	6/6	6/6	6/6	6/6	0/6	6/6	6/6	6/6	6/6
**500**	4/6	6/6	6/6	6/6	6/6	6/6	0/6	4/6	6/6	6/6	0/6
**50**	0/6	6/6	6/6	0/6	6/6	6/6	0/6	1/6	6/6	26	0/6

**TRIAL 2**											
**0**	0/6	0/6	0/6	0/6	0/6	0/6	0/6	0/6	0/6	0/6	0/6
**13,000**	6/6	6/6	6/6	6/6	6/6	6/6	1/6	6/6	6/6	6/6	6/6
**1300**	6/6	6/6	6/6	2/6	1/6	6/6	0/6	6/6	6/6	6/6	0/6
**130**	3/6	6/6	6/6	0/6	0/6	6/6	0/6	2/6	4/6	2/6	0/6
**13**	1/6	6/6	6/6	0/6	0/5***	4/5***	0/6	0/6	4/6	0/5 ***	0/5***

**TRIAL 3**											
**0**	0/6	0/6	0/6	0/6	0/6	0/6	0/6	0/6	0/6	0/6	0/6
**8000**	6/6	6/6	6/6	6/6	0/6	6/6	0/6	6/6	6/6	6/6	5/6
**800**	6/6	6/6	6/6	0/6	0/6	6/6	0/6	6/6	6/6	6/6	4/6
**80**	6/6	6/6	6/6	0/6	0/6	6/6	0/6	6/6	6/6	6/6	1/6
**8**	6/6	6/6	6/6	0/6	0/6	6/6	0/6	0/6	6/6	0/6	0/6

TP*	50	68	68	26	25	64	1	43	62	50	40
TN*	18	18	18	18	18	18	18	18	18	18	18
FP*	0	0	0	0	0	0	0	0	0	0	0
FN*	16	0	0	40	40	1	65	23	4	15	26

AC**	0.8	1.0	1.0	0.52	0.52	0.99	0.23	0.73	0.95	0.82	0.69
SE**	0.76	1.0	1.0	0.4	0.38	0.98	0.02	0.65	0.94	0.77	0,61
SP**	1.0	1.0	1.0	1.0	1.0	1.0	1.0	1.0	1.0	1.0	1.0

*TP = True positives, **TN **= True negatives, **FP **= False positives, **FN **= False negatives

**AC = Accuracy, **SE **= Sensitivity, **SP **= Specificity

*** Due to a technical fault, one of the sextuplicates in the lowest spiking level was excluded for the Selecta enrichment based methods.

**Table 4:** Results from trial 4–6; pig faecal samples artificially contaminated with salmonella. Faeces originating from three pig farms. For each trial different spiking levels of salmonella were constructed (Trial 4 *S*. Derby, Trial 5 *S*. Cubana, Trial 6 *S*. Typhimurium DT40), each spiking level containing six 25 g samples of faeces.

**CFU salm/25 g**	**NMKL**	**MSRV**	**MSRV PCR**	**VIDAS SLM**	**SELECTA ELISA**	**SELECTA CULTURE**	**OPTIMA ELISA**	**OPTIMA CULTURE**	**BAX^®^**	**SELECTA PCR**	**SELECTA IMS PCR**
**TRIAL 4**											
**0**	0/6	0/6	0/6	0/6	0/6	0/6	0/6	0/6	0/6	0/6	0/6
**100,000**	6/6	6/6	6/6	6/6	6/6	6/6	6/6	6/6	6/6	6/6	6/6
**10,000**	6/6	6/6	6/6	6/6	6/6	6/6	6/6	6/6	6/6	6/6	6/6
**1000**	3/6	6/6	6/6	6/6	6/6	6/6	4/6	6/6	6/6	6/6	6/6
**100**	0/6	6/6	6/6	3/6	6/6	6/6	0/6	4/6	6/6	6/6	6/6
**10**	0/6	6/6	6/6	5/6	5/6	4/6	0/6	0/6	6/6	6/6	6/6

**TRIAL 5**											
**0**	0/6	0/6	0/6	0/6	0/6	0/6	0/6	0/6	0/6	0/6	0/6
**12,000**	6/6	6/6	6/6	6/6	6/6	6/6	3/6	6/6	6/6	6/6	6/6
**1200**	6/6	6/6	6/6	6/6	6/6	6/6	1/6	2/6	6/6	6/6	6/6
**120**	5/6	6/6	6/6	5/6	4/6	6/6	0/6	0/6	6/6	6/6	6/6
**12**	1/6	6/6	6/6	0/6	1/6	6/6	0/6	0/6	4/6	6/6	5/6

**TRIAL 6**											
**0**	0/6	0/6	0/6	0/6	0/6	0/6	0/6	0/6	0/6	0/6	0/6
**430,000**	6/6	6/6	6/6	6/6	6/6	6/6	6/6	6/6	6/6	6/6	6/6
**38,000**	0/6	6/6	6/6	5/6	2/6	5/6	0/6	0/6	5/6	6/6	6/6
**3,800**	0/6	6/6	6/6	0/6	0/6	1/6	0/6	0/6	6/6	2/6	3/6
**380**	0/6	6/6	6/6	0/6	0/6	0/6	0/6	0/6	6/6	0/6	1/6
**38**	0/6	2/6	2/6	0/6	0/6	0/6	0/6	0/6	5/6	0/6	0/6

TP*	38	80	80	54	54	54	26	36	80	68	69
TN*	18	18	18	18	18	18	18	18	18	18	18
FP*	0	0	0	0	0	0	0	0	0	0	0
FN*	46	4	4	30	30	30	58	48	4	15	15

AC**	0.54	0.96	0.96	0.71	0.71	0.71	0.43	0.53	0.96	0.82	0.85
SE**	0.45	0.95	0.95	0.64	0.64	0.64	0.31	0.42	0.95	0.77	0.82
SP**	1.0	1.0	1.0	1.0	1.0	1.0	1.0	1.0	1.0	1.0	1.0

*TP = True positives, **TN **= True negatives, **FP **= False positives, **FN **= False negatives

**AC = Accuracy, **SE **= Sensitivity, **SP **= Specificity

**Table 5:** Results from trial 7–9; cattle faecal samples artificially contaminated with salmonella. Faeces originating from one cattle farm (Trial 7) and three cattle farms (Trial 8–9). For each trial different spiking levels of salmonella were constructed (Trial 7 *S*. Typimurium DT1, Trial 8 *S*. Dublin, Trial 9 *S*. Tennesse), each spiking level containing six 25 g samples of faeces

**CFU salm/25 g**	**NMKL**	**MSRV**	**MSRV PCR**	**SELENIT CYSTINE**	**VIDAS SLM**	**SELECTA ELISA**	**SELECTA CULTURE**	**OPTIMA ELISA**	**OPTIMA CULTURE**	**BAX^®^**	**SELECTA PCR**	**SELECTA IMS PCR**
**TRIAL 7**												
**0**	0/6	0/6	0/6	0/6	0/6	0/6	0/6	0/6	0/6	0/6	0/6	0/6
**15,000**	6/6	6/6	6/6	6/6	6/6	6/6	6/6	6/6	6/6	6/6	6/6	6/6
**1,500**	6/6	6/6	6/6	6/6	6/6	6/6	6/6	6/6	6/6	6/6	6/6	6/6
**150**	6/6	6/6	6/6	6/6	0/6	6/6	6/6	0/6	3/6	6/6	6/6	6/6
**15**	3/6	6/6	6/6	6/6	0/6	6/6	6/6	0/6	0/6	6/6	6/6	6/6

**TRIAL 8**												
**0**	0/6	0/6	0/6	0/6	0/6	0/6	0/6	0/6	0/6	0/6	0/6	0/6
**400,000**	2/6	N.P***	N.P***	6/6	3/6	N.P***	N.P***	N.P***	N.P***	N.P***	N.P***	N.P***
**82,000**	0/6	6/6	6/6	6/6	0/6	6/6	6/6	0/6	0/6	N.P***	N.P***	N.P***
**9,000**	0/6	6/6	6/6	2/6	0/6	6/6	6/6	0/6	0/6	6/6	6/6	6/6
**900**	0/6	6/6	6/6	0/6	0/6	6/6	6/6	0/6	0/6	0/6	6/6	6/6
**90**	0/6	6/6	6/6	0/6	0/6	1/6	4/6	0/6	0/6	0/6	6/6	6/6
**9**	0/6	5/6	5/6	0/6	0/6	0/6	2/6	0/6	0/6	0/6	1/6	3/6

**TRIAL 9**												
**0**	0/6	0/6	0/6	0/6	0/6	0/6	0/6	0/6	0/6	0/6	0/6	0/6
**6,600**	6/6	6/6	6/6	5/6	6/6	6/6	6/6	5/6	6/6	6/6	6/6	6/6
**660**	6/6	6/6	6/6	6/6	6/6	6/6	6/6	5/6	6/6	6/6	6/6	6/6
**66**	2/6	6/6	6/6	3/6	0/6	6/6	6/6	1/6	6/6	6/6	6/6	6/6
**7**	0/6	6/6	6/6	1/6	0/6	2/6	6/6	0/6	0/6	6/6	6/6	6/6

TP*	37	77	77	53	27	63	72	23	33	54	67	69
TN*	18	18	18	18	18	18	18	18	18	18	18	18
FP*	0	0	0	0	0	0	0	0	0	0	0	0
FN*	47	1	1	31	57	15	6	55	45	18	5	3

AC**	0.54	0.99	0.99	0.69	0.44	0.84	0.94	0.43	0.53	0.8	0.94	0.97
SE**	0.44	0.99	0.99	0.63	0.32	0.81	0.92	0.29	0.42	0.75	0.93	0.96
SP**	1.0	1.0	1.0	1.0	1.0	1.0	1.0	1.0	1.0	1.0	1.0	1.0

*TP = True positives, **TN **= True negatives, **FP **= False positives, **FN **= False negatives

**AC = Accuracy, **SE **= Sensitivity, **SP **= Specificity

***N.P = not performed

### Results from the pooled samples consisting of assumed salmonella- free faeces

The results from the analyses of the 100 suspected salmonella-negative pooled faecal samples are summarized in Table 2. Except for the BAX^® ^protocol, which indicated one positive sample, none of the other methods revealed salmonella in any of the 100 "unspiked samples". Three methods (Vidas SLM, Bioline Optima and Bioline Optima culture) failed to detect salmonella in one of the spiked control samples that were run parallel with the unspiked samples.

### Results of Trials 1–3: poultry faeces spiked with different concentrations of salmonella

The results from trial 1–3 are presented in Table 3.

In Trial 1, mixed faeces from two egg-producing farms were spiked with different concentrations of *S*. Enteritidis.

In Trial 2, mixed faeces from two egg-producing farms and one broiler farm were spiked with different levels of *S*. Worthington. Due to a technical fault, one of the sextuplicates in the lowest spiking level was excluded from the Selecta enrichment based methods. Only the MSRV-based methods succeeded in detecting salmonella in all spiked samples (SE = 1.0). The methods involving any immunological technique (Vidas SLM, Bioline Optima, Bioline Selecta and Selecta- IMS- PCR) were in this trial generally less sensitive than the methods based on culturing only or PCR without any immunological techniques involved.

In Trial 3, mixed faeces from two egg-producing farms and one broiler farm were spiked with different concentrations of *S*. Livingstone. As in Trial 2 the ELISA-based methods in this trial were generally less sensitive than the other method. Bioline Selecta failed to detect salmonella in any of the spiked samples whereas culturing with the Selecta enrichment found salmonella in all the spiked samples. The Selecta- IMS-PCR that included an immunological technique (IMS) had on the other hand in this trial a sensitivity of 0.67, almost equal to Selecta-PCR (SE = 0.75).

### Results from Trials 4–6: pig faeces spiked with different concentrations of salmonella

The results from trial 4–6 are presented in Table 4.

In Trial 4, mixed faeces from three pig farms were spiked with different concentrations of *S*. Derby. The NMKL methods, NMKL (SE = 0.50) and Bioline Optima culture (SE = 0.73) were in this trial relatively less sensitive than the other methods.

In Trial 5, mixed faeces from three pig farms were spiked with different concentrations of S. Cubana while in Trial 6 similarly S. Typhimurium DT40 was used. In trail 6, all culturing methods had problems in detecting this S. Typhimurium DT40 strain at the lower spiking levels. The MSRV method failed to detect salmonella in 4 samples at the lowest level (SE = 0.87). Whereas throughout the study the MSRV method detected all the positive salmonella samples after 24 h incubation of the MSRV agar plates, in this trial, 5 of the 26 positive samples were not detected until the MSRV agar plates had been incubated for 48 h. For one sample, at the lowest inoculation level, plating out from the MSRV did not yield any salmonella colonies even though MSRV-PCR confirmation yielded a positive result. After a new attempt to plate out from the MSRV agar plate, positive salmonella colonies were detected.

The NMKL methods (NMKL and Bioline Optima culture) required spiking concentrations as high as 430 000 cfu/25 g before they were able to detect salmonella in any sample. The Bioline Selecta culture method also had problems and required spiking concentrations as high as 3800 cfu/25 g until the method was able to detect any positive sample.

The BAX^® ^PCR protocol (SE= 0.93) had best sensitivity of all methods in Trial 6. The in-house PCR methods based on the Bioline Selecta enrichment protocol had sensitivities ranging between 0.47 and 0.53

### Results from Trials 7–9: cattle faeces spiked with different concentrations of salmonella

The results from trial 7–9 are presented in Table 5. Trials 7–9 included, in addition to the methods used in Trial 1–6, the Selenite/Cystine method.

In Trial 7, mixed faeces from one farm were spiked with different concentrations of S. Typhimurium DT1.

In Trial 8, mixed faeces from three cattle farms were spiked with different concentrations of S. Dublin strain. In this trial all methods based on RVS enrichment had difficulties in detecting this particular S. Dublin strain. As none of the RVS enrichment based methods detected any positive samples in the first test run at the highest spiking level of 9000 cfu/25 g, a re-run was performed with higher spiking concentrations (82,000 cfu and 400,000 cfu/25 g faeces). However, analyses in this re-run were not performed for all methods. The RVS enrichment based NMKL method was unable to detect any positive salmonella at any spiking level lower than 400,000 cfu/25 g faeces (SE = 0.06) whereas MSRV based methods (SE = 0.97), Bioline Selecta culture (SE = 0.80) and PCR methods based on Bioline Selecta enrichment broth (SE = 0.87 and 0.79 respectively) detected salmonella in the trials lowest spiking level (9 cfu/25 g faeces). The BAX^® ^method did not detect any positive sample at any level lower than 9000 cfu/25 g faeces. The Selenite/Cystine method sensitivity (SE = 0.39) surpassed the RVS enrichment based methods.

In Trial 9, mixed faeces from three cattle farms were spiked with different concentrations of a S. Tennnesse strain.

### Extended method for isolation of salmonella from the samples indicated to be salmonella-positive by the BAX^® ^salmonella detection method

The BAX^® ^system produced no positive results from the non-seeded control samples in the nine trial runs. It did, however, yield one suspected response from the 100 pooled faecal samples that were believed to be free from salmonella (consisting of faeces from the 265 assumed salmonella free farms). From this sample we attempted to isolate salmonella, as described in Materials & Methods, but were unsuccessful when using the additional methods. Nor was this sample found salmonella positive by any other salmonella detection method, including culture-based, ELISA and in-house PCR methods. We therefore chose to regard this sample as a false positive result

## Discussion

Overall, the MSRV method proved to be the most reliable and sensitive method for detecting salmonella in faecal samples, regardless of which species the faeces originated from. The MSRV method requires the selective agar plates to be read after both 24 and 48 hours. In our study, only one trial run yielded more positive samples after 48 h than after 24 h incubation of the MSRV agar plates. These were the samples spiked with S. Typhimurium DT40, which also proved difficult to detect with several other salmonella detection methods used in the study. A 48 h incubation of MSRV agar plates has been shown to increase the number of detected salmonella-positive samples in other studies [3,4,6]. To hasten the confirmation of suspected salmonella growth on MSRV, a PCR-based colony confirmation technique was used directly from the MSRV agar plates, which proved to be rapid, specific and sensitive. The PCR confirmation succeeded in detecting all the MSRV-positive samples in the study, and in addition one extra positive sample which was not detected by conventional confirmation, but which was shown to be a true positive by a second attempt to isolate salmonella from the MSRV agar plate.

The PCR-based methods used in the study proved almost as sensitive and specific as the MSRV method. However, the ELISA-based methods performed less well, and this seemed to be correlated to the salmonella strain used when spiking the faecal samples in the different trials. For instance, S. Livingstone and S. Worthington were not easily detected by some of the ELISA methods used, probably because of poor binding to the antibodies. This was also noted when using immuno magnetic separation (IMS) for salmonella with Dynal magnetic beads coated with antibodies, and is a clear drawback for this detection strategy.

The first set of samples analysed was 100 assumed salmonella-negative pooled samples consisting of faeces originating from 265 different Swedish farms. These samples were so devised as to have a wide variation in the composition of the intrinsic bacterial flora. This was done in order to attempt to elicit false positive results by the different salmonella detection methods used. This could be a problem especially for methods using antibody-based salmonella detection strategies, if the antibodies are not sufficiently specific.

In the seeding experiments, spiked samples were prepared, divided among nine different trial sets; three each using poultry, pig and cattle faeces. As the experiment was performed in Sweden, and naturally infected faecal samples are rare and difficult to obtain, artificially contaminated samples were used. To attempt to imitate natural conditions, where salmonella bacteria might be stressed, the salmonella cultures were subjected to stress treatments before seeding them into the faecal samples. We also tried to prepare the different spiking concentrations so that the NMKL method would be able to detect salmonella in about half of the spiked samples in each trial set, as our aim was to evaluate the possibility of replacing the NMKL method with a more rapid salmonella detection method.

The very low levels of stressed salmonella bacteria, as seen in the lowest spikning level in our study, might not be so frequently encountered among naturally infected samples. Thus in our opinion, if a method in the present study performs with sensitivity twice as high as any other method, this does not necessarily mean that the method would reveal twice as many salmonella-positive samples in naturally infected faeces. What it does imply is that the method is more likely to detect salmonella in faeces when only a small number of stressed salmonella bacteria are present, and where the intrinsic flora is abundant and varied. This might for instance be the case when analysing pooled faecal samples.

It is difficult to compare our results with other studies, where other reference methods may have been used. Also, in other studies, different enrichment protocols and media may have been used and there may have been slight differences in the protocols used. Furthermore, our pretreatment to stress the salmonella bacteria that was used in the trials and the treatment of the spiked samples may have influenced the results.

### The NMKL method (no71, version 5)

The low sensitivity obtained with S. Typhimurium DT40 and S. Dublin made the NMKL methods perform relatively poorly in our study. Even if we disregard the results regarding these two serotypes, the NMKL methods are generally less sensitive than the MSRV-method and the Selecta culturing method (Table 1). These results are consistent with other studies [3,4] that have indicated that MSRV enrichment is more sensitive than RVS enrichment.

The NMKL method and the ISO 6579 method are currently the methods that are approved for use for faecal samples in the Swedish salmonella control program (SCP). In our study, these were the reference method with which all other methods were compared. The NMKL method showed a variable ability to detect salmonella in the spiked faecal samples, depending on the strain used. The method was performed as two separate analyses run in parallel. One analysis used RVS enrichment broth manufactured by Oxoid and the other RVS broth that was supplied by Bioline. In both cases the NMKL method had severe problems in detecting 2 of the 9 different salmonella strains used, viz. S. Typhimurium DT40 (St 506/04; seeded in pig faces in Trial set 6) and S. Dublin (St 31/04; seeded in cattle faces in Trial set 8). The salmonella strains used in our study were chosen to be typical serotypes found in the routine diagnostics of respective animal species, and S. Typhimurium DT40 is the serotype most commonly encountered in pigs in the SCP, particularly in mesenteric lymph nodes from slaughtered pigs. S. Dublin, the other serotype that caused problems in the NKLM method, is one of the most commonly found serotype in cattle faeces.

In a separate study performed at The National Veterinary Institute we analysed 265 faecal samples collected from cattle in a S. Dublin infected herd, using the conventional NMKL method, the Selenite/Cystine method and the MSRV method as described in this study. Of a total of 34 samples proven positive, 31 were found using the MSRV-method, 26 by Selenite/Cystine and only 9 by conventional NMKL method (data not shown). This tallies with the results presented in the present study.

The strain that was used to spike the cattle feacal samples in our study was isolated from a farm situated in the same part of Sweden as the above-mentioned S. Dublin infected herd. It may be that both farms were infected with the same salmonella strain and that the NMKL method in particular has problems in detecting this specific strain. The sensitivity for other S. Dublin strains may be better.

The low degree of sensitivity obtained with S. Typhimurium DT40 and S. Dublin made the NMKL methods perform relatively poorly in the study. Even if one disregards the results obtained with these two serotypes, the overall sensitivity of the NMKL methods is less than with both the MSRV method and the Selecta culturing method (Table 1). Our results are consistent with those of the EU's CRL for salmonella [3,4].

### The MSRV method

This method is to be incorporated into an Annex to the ISO method 6579 for salmonella detection, and is the method recommended by EU's CRL for analysis of faecal samples from animals [12]. It was included in our study as it was deemed important to compare the performance of this method with the NMKL method currently used in the SCP.

In our study, the MSRV method was the most sensitive and specific method. It detected all the nine different salmonella strains used, at very low spiking levels. It performed equally well with faeces from the three animal species included in the study. In addition to the standard confirmation protocol from MSRV agar plates (see Material and Methods) we used real-time PCR to quickly confirm the presence of salmonella in the growth zones on the MSRV-agar plates. One loop 1 μl was dipped into the zone and colony material was transferred to a pre-prepared PCR-tube and the PCR analysis was performed immediately. This 1-day speed-up of the confirmation can be advantageous in, for instance, the event of a salmonella outbreak situation. This quicker PCR confirmation performed equally well as the standard confirmation protocol.

### Enrichment according to the Bioline Selecta protocol, confirmed by cultivation on selective agar plates

For salmonella detection the Selecta protocol with enrichment and subsequent cultivation on XLD- and BGA agar plates is 1-day quicker than the NMKL method. The overall calculated performance of the Selecta culturing method gave a sensitivity of 0.88 compared with the NMKL method, which showed a sensitivity of 0.54 (Table 1). However, a drawback with the Selecta protocol is that it is difficult to handle large sample volumes as it is of uttermost importance to maintain the appropriate temperatures as well as to comply strictly to the incubation times in the different enrichment steps, as recommended by the manufacturer.

When evaluating results obtained with the Selecta enrichment protocol one should take into account that the incubation times used in this study were not optimal (for the Selecta enrichment). The manufacturer recommends pre-enrichment (in pre-warmed BPW) for 6–10 h and enrichment (with pre-warmed Selecta broth) for 18–24 h. In our study, in order to test the method's capacity to provide results quickly, BPW pre-enrichment was performed for 6–7 h and Selecta enrichment for 18–19 h. If these incubation times had been extended, performance using the methods based on Selecta enrichment may well have been improved.

### Bioline Selecta

The Bioline Selecta method is a commercially available assay for salmonella detection, based on a patented enrichment protocol completed within 26 h, and subsequent analysis with an ELISA kit. The method has been validated according to NORDVAL [13] and AFNOR criteria [14]. In Sweden it is approved by the Swedish National Food Administration (NFA) for salmonella detection within the official national control program for food.

Bioline Selecta is one of the quickest salmonella detection methods included in this study. Compared with the NMKL methods, Bioline Selecta ELISA gave better results for both porcine and bovine faecal samples (Tables 4 and 5). With the poultry samples, however, some difficulty was encountered in Trial 2 and especially in Trial 3, possibly due to matrix problems when broiler faeces were mixed with faeces from layer hens in these two trials. Another plausible explanation is that the antibodies used in the ELISA had difficulties in detecting the specific strains of salmonella used in these trial sets (S. Worthington and S. Livingstone). Such problems may be related not to the serotypes but to these particular salmonella strains. Most probably these strains were spread to Swedish poultry farms by dry feedstuffs and may have undergone some alteration in immunological composition during storage. This finding warrants further investigation.

### Bioline Optima

Bioline OPTIMA is a commercially available assay for salmonella detection, where a pre-enrichment step using BPW and a selective enrichment step with RVS broth preceede salmonella detection with an ELISA kit. The method has been validated for food samples according to NORDVAL [13] and AFNOR criteria [14]. In Sweden it is sanctioned by the NFA for salmonella detection within the official national control program for food. The Bioline Optima ELISA method showed the poorest performance of all methods included in the study, with an overall calculated sensitivity of 0.23 (see Table 1). Performance was especially poor for the poultry samples (SE = 0.02) whereas the trials with porcine and bovine faeces gave somewhat better results (SE = 0.31 and 0.29, respectively). Bioline Optima culturing method gave results generally comparable to the NMKL method (SE = 0.49 versus SE= 0.54), results that are reasonable since the only difference between the methods was that we used RVS enrichment broth from different producers.

### VIDAS SLM

Vidas SLM, a commercially available method, is currently used by regional microbiological laboratories in Sweden, and was therefore included in the study. The method has been validated for food samples according to NORDVAL [13] and AFNOR criteria [14]. In Sweden it is sanctioned by the NFA or salmonella detection within the official national control program for food.

Several enrichment protocols for salmonella (for different matrices) were available for the VIDAS system when the study was performed in 2004. However, after discussion with the manufacturer we agreed to use no other enrichment broths than RVS and M-broth. The protocol used was also the enrichment protocol recommended by the manufacturer at the time. Another reason for leaving out other enrichment broths was that we wanted to evaluate the performance of the VIDAS system on faecal samples using a protocol based solely on the same enrichment broths that were routinely used by Swedish regional laboratories when analysing food samples.

The enrichment protocol used in the present study, as was recommended by the manufacturer, might not be optimal for faecal samples. In a collaboration initiated after the study, the manufacturer has repeated some of the experiments using a modified enrichment protocol, using BPW followed by MKTTn and M broth, which produced considerably improved results.

In our study, the Vidas SLM method performed comparably to the NMKL method (Table 1), giving slightly better results for pig faeces and slightly poorer results for cattle and poultry faeces. These results are consistent with the study performed by Korsak et al. [7] who found that the Vidas system was comparable to the NMKL71 method. In another study Dam-Deisz et al.[5] concluded that Vidas gave better results for cattle samples than the compared culture method, though the reference culture method in that study included only a single enrichment step and cannot be compared with the reference methods used in our study.

The poor results for S. Dublin in cattle faeces may well be due to the same poor performance with enrichment broths as was observed with the NMKL method. As for the poultry samples the Vidas SLM performed badly regarding S. Livingstone, possibly due to the poor binding of specific salmonella strains to the antibodies used in the ELISA kit, similar to the findings for Selecta-ELISA described above.

### BAX^® ^(PCR method)

BAX^® ^system worked well in the trial, except with the S. Dublin samples in Trial 8. In all other trial sets the sensitivity of the BAX^® ^system was excellent. The lower sensitivity in Trial 8 could in our opinion have been because the S. Dublin strain used did not grow so well in the second enrichment medium (TT-Hajna) used in the BAX^® ^protocol, modified for optimal performance in faecal samples. This replicates the poor growth of S. Dublin in methods depending on RVS broth and Selenite/Cystine for secondary enrichment.

In an American study, performance with the BAX^® ^system in feed analysis was compared with a most probable number (MPN) method [15]. That study revealed the sensitivity of the BAX^® ^system to be dependent on the enrichment protocol applied. As in our study a modified protocol, recommended by the manufacturer, was used to reduce the risk of PCR inhibition. It was also shown that sensitivity improved when enrichment was prolonged from 24 h to 48 h.

BAX^® ^identified one sample in the set of 100 un-seeded pooled samples as positive for salmonella. At the time of the study, the protocol used for BAX^® ^was a draft protocol, as suggested by the manufacturer, and the protocol we received did not include any specified confirmation step. Therefore, no confirmation step for the BAX^® ^method was used. However, none of the other methods could verify this sample as salmonella positive. Neither could further attempts with repeated analyses by culture methods, performed on stored material, confirm this sample as salmonella positive.

It might be that the BAX^® ^system detected an atypical salmonella strain that was difficult to isolate by the culturing methods used.

It seems unlikely that DNA from dead salmonella bacteria present in the faecal sample could explain this positive result. If such DNA had been present in the pooled faecal sample, the two enrichment steps must have considerably lowered the concentration of DNA, available for PCR detection. Furthermore, none of the other included PCR method, which also would have been able to detect DNA from dead salmonella bacteria, classified this sample as positive.

However, as we were unable to confirm the positive salmonella finding this result was considered as a false positive and the overall specificity was therefore calculated to be 0.99.

### Bioline Selecta followed by in-house PCR

By using PCR as confirmation after the Bioline Selecta protocol, a rapid method was obtained, the most rapid method together with Bioline Selecta Elisa of the methods tested in the study. The method had a sensitivity of 0.88 and no false positive results were obtained among the 100 non-seeded samples. This is a very easy method to perform, as no DNA extraction was done before the PCR analysis. Also, this would be a very low-cost screening method. The method may further have potential to improve its sensitivity if the incubation times in the Selekta enrichment protocol is extended see discussion above (Selecta culturing method).

However, the method needs to be validated to be able to use it for official Salmonella control.

### Bioline Selecta enrichment followed by IMS separation of salmonella and in-house PCR

To try to enhance the sensitivity of the in-house PCR we introduced an immuno-magnetic separation step, using salmonella-specific antibodies prior to the PCR analysis. The immuno-magnetic step makes this method take a little longer time than the method described above. It will also be more expensive to perform, as the immuno-magnetic beads with antibodies to salmonella are quite expensive, and as we also included a DNA-separation step before PCR, because using the beads directly in the PCR strongly inhibited the PCR reactions. In spite of adding the immuno-magnetic and the DNA extraction steps, this method does not perform better than Bioline Selecta enrichment followed by in-house PCR as described above. We believe that the method performs differently, depending on the salmonella strain used for seeding, because some serotypes/strains bind weakly to the antibodies used. When using salmonella serotypes/strains having a high affinity to the immuno-magnetic beads, a high degree of sensitivity is obtained, but for other serotypes/strains the method does not perform equally well.

### Summary of results of PCR- based methods used in the study

In a minireview article, Malorny and Hoorfar [16] discuss different approaches to diagnostic PCR testing of porcine faecal samples. Optimal enrichment is important, as it must enable sub-lethally injured salmonella cells to recover and grow but simultaneously should inhibit the growth of the background flora. The PCR protocols must be modified to prevent the problem of PCR inhibition that occurs when using faecal samples. The authors also discuss the potential of PCR methods for quantification of salmonella in pig faeces.

In a Canadian study [10] faecal samples from 67 pigs were tested with microbiological culture and a cultivation PCR assay. The authors concluded that their PCR assay had a low detection limit and detected similar numbers of shedding and non-shedding animals as did the culture method. The PCR assay in that study was performed on a mixture of three different enrichment broths incubated for 5 days. An American study [9] in 1994 also presented results showing that their combined cultivation PCR assay identified salmonella serovars in clinical samples from pigs, horses and cattle with same sensitivity and specificity, but more quickly than with the conventional culture techniques being compared.

On the whole, the PCR based methods in our study were very sensitive, especially when compared with the NMKL method. Some are comparable to the MSRV method, which was the most sensitive method of all. However, a possible drawback of PCR methods is that they may yield positive results for salmonella, but confirmation by culturing cannot be obtained. This could be due to stressed bacteria or because certain serotypes are difficult to culture, but also to false positive results of the PCR-based method. If, on the other hand, these methods (and likewise the ELISA methods) are regarded as a screening test, where positive signals need to be confirmed by culturing techniques, this would be acceptable. If a negative PCR result is obtained, the analysis can be terminated, and further confirmation is unnecessary. The most important feature of a method used as a screening test is its sensitivity, and a lower specificity is acceptable. However, when too many samples test positive and cannot be confirmed by culturing, the method become too laborious

## Conclusion

The MSRV method was the method in our study that performed with greatest specificity and sensitivity. It could detect very low concentrations of spiked, stressed salmonella bacteria of different serotypes and in faecal samples from different animal species. The NMKL method, which is currently used in the SCP, did vary substantially in performance; depending on which salmonella strain was seeded into the faecal samples.

The PCR-based salmonella detection methods used in our study performed almost as well as the MSRV method, though the ELISA-based methods were serotype or strain dependent, and did not give a satisfactory overall performance.

## Methods

### Pooled faecal samples presumed to be salmonella-free

From 265 randomly selected farms, one faecal sample from each farm was collected, faeces deriving from different places in the stables. Samples of bovine faeces were collected by staff of the Swedish Life Stock Associations. Porcine faecal samples were collected by staff of the Swedish Animal Health Service, and faecal samples from poultry were kindly provided by several poultry farmers. All samples were sent anonymously to the National Veterinary Institute (NVI).

At NVI the 265 faecal samples were used to prepare 100 pooled samples (see Table 2). When preparing the pooled samples, faeces from 1–3 farms (faeces from the same species) were placed in a jar. Faeces were thoroughly mixed whereafter the pooled sample was constructed by transferring 25 g of the mixed faeces into a new jar. Eleven pooled faecal samples were constructed from broilers farms (faeces from 33 farms); 23 from laying hens farms (faeces from 43 farms); 33 from pigs farms (faeces from 98 farms) and 33 from cattle farms (faeces from 91 farms).

For the first set of samples (samples 1–33, see Table 2) faeces were collected on a Thursday and sent by ordinary regular mail without a cooling device. On the following day the samples were pooled and left at 4–8°C until the Monday, when the analysis for salmonella by the different methods described below was commenced.

For the second set of samples (samples 34–66) faeces were collected and mailed as above, but after pooling they were left at room temperature until the Monday when salmonella analysis was performed. For the third set (samples 67–100) faeces were sent to the lab on a Monday by regular mail without a cooling device. The samples were pooled on the Tuesday whereafter salmonella analysis was commenced. One salmonella- positive sample was included in each of the three sets, prepared by seeding one faecal sample with a different strain of salmonella for each set (see Table 6).

**Table 6:** Salmonella strains used in the different trials

**STRAIN ID**	**SEROVAR SALMONELLA**	**ORIGIN**	**TRIAL**
St 784/02	*S*. Enteritidis	Faecal sample, goose	Trial 1
St 332/04	*S*. Worthington	Faecal sample, duck	Trial 2 Positive control strain*
St 370/03	*S*. Livingstone	Faecal sample, egglaying hens	Trial 3
St 1130/98	*S*. Derby	Faecal sample, swine	Trial 4
St 830/03	*S*. Cubana	Faecal sample, swine	Trial 5
St 506/04	*S*. Typhimurium DT40	Mesenteric lymph node, swine	Trial 6
St 77/02	*S*. Typhimurium DT1	Faecal sample, cattle	Trial 7 Positive control strain*
St 31/04	*S*. Dublin	Faecal sample, cattle	Trial 8
St 998/03	*S*. Tennessee	Mesenteric lymph node, cattle	Trial 9
St 327/00	*S*. Typhimurium DT40	Faecal sample, swine	Positive control strain*

* these strains were used as a positive control strain in one sample each when analysing assumed salmonella-free faecal samples

As the presence of salmonella among Swedish food-producing animals is extremely low, the pooled samples were assumed to be free of salmonella. The intention was to obtain a heterogeneous bacterial flora in these samples, so as to elicit possible false positive results when evaluating the different rapid methods for salmonella detection.

### Spiked faecal samples

Nine separate sets of trials with spiked faecal samples were performed, each set using fresh faecal samples and different strains of salmonella (see Tables 3, 4, 5). The faeces used derived from 9 farms (3 poultry farms, 3 pig farms and 3 cattle farm).

At each farm, faeces were collected from different age categories. For each animal category, faeces were sent in from the same farms throughout the different trial sets. However for each new trial, a new set of fresh faeces were collected.

The salmonella strains used represented serotypes commonly isolated at routine faecal sampling in Sweden.

For each set, faeces from up to three different farms, were carefully homogenized in a bucket. Trial 1 comprised faeces from two laying poultry farms whereas Trials 2 and 3 included faeces from two laying poultry farms and a broiler farm. Faeces in Trials 4, 5 and 6 comprised faeces from three pig farms. In Trial 7 faeces from only one cattle farm were used whereas in Trials 8 and 9 the faeces were obtained from three cattle farms.

After the faeces had been homogenized, faecal samples in of 25 g aliquots were seeded with salmonella bacteria of different concentrations, preparing six replicates for each concentration. The number of different concentrations used for seeding the faecal samples varied between the different trial sets. For each trial set, six non-seeded samples were analysed as negative controls (see Tables 3, 4, 5)

The salmonella strains were first grown on brom-cresol-purpure-lactose agar plates (blue-agar) (Oxoid, Basingstoke, England). For each trial, on a Thursday, one colony was inoculated into 5 ml Nutrition broth supplemented with 10% bovine serum (In house, National Veterinary Institute), which was then incubated at 37°C for 18–24 h. The next day, Friday, serial dilutions [1,10] in peptone saline water were prepared from the serum broth. These dilution tubes were kept at 2–4°C until Monday morning when the prepared faecal samples were spiked by adding aliquots of peptone saline water from selected dilution tubes. The intention was to stress the salmonella bacteria and to secure that the added bacteria should not be in growth phase when the faecal samples were spiked. Thereafter, the salmonella analysis commenced, according to the methods described below. In parallel, the added numbers (cfu salmonella) were estimated by plating out peptone saline water from selected dilution tubes on blue agar plates. These agar plates were incubated at 37°C 18–24 h whereafter cfu were counted.

Altogether 288 spiked samples were constructed in the study. However, all spiked samples were not analysed by all methods.

The Selenite/Cystine protocol was only used in Trials 7–9 with cattle faeces (Table 5). In Trial 2 (S. Worthington in poultry faeces) one of the sextuplicates from the lowest inoculation concentration (13 cfu/25 g faeces) was excluded from methods that utilized the Bioline Selecta enrichment protocol (Bioline Selecta (ELISA), Bioline Selecta culture, Selecta-PCR and Selecta-IMS-PCR) due to a technical error during the enrichment procedure (see Table 3).

In Trial 8 (S. Dublin in cattle faeces) analyses were not performed for the sextuplicates from the highest inoculation concentration 400,000 cfu/25 g faeces) for the methods MSRV, MSRV-PCR, Bioline Selecta (ELISA), Bioline Selecta culture, Bioline Optima (ELISA), Bioline Optima culture, BAX^® ^system, Selecta -PCR and the Selecta-IMS-PCR. Furthermore in this trial analyses were not performed for sextuplicates in the second highest inoculation concentration (82,000 cfu/25 g) for the PCR methods BAX^®^, Selecta PCR and Selecta-IMS-PCR (see Table 5)

### Bacterial strains

The salmonella strains used in the study are presented in Table 6. All were isolated from faeces or mesenteric lymph from Swedish livestock. They were serotyped at the National Veterinary Institute, Uppsala, and, when appropriate, phage-typed by the Swedish Institute of Infectious Disease Control, Stockholm. Subsequently they have been kept at -70°C. Before use in this study, they were grown on non-selective agar plates (blue agar) and then used to prepare dilution series as described above.

### Pre-enrichment in buffered peptonewater (BPW)

For all methods the first step in the analyses was pre-enrichment of 25 g faeces in 225 ml Buffered Peptone Water (BPW (CM 0509) (Oxiod, Basingstoke, England).

In order to perform all salmonella procedures according to each manufacturer's instructions, two parallel sets of samples had to be pre-enriched in BPW for each trial set.

The first pre-enrichment set was started at 8 am using BPW pre-warmed to 37°C. This set was used for the methods based on the Selecta enrichment protocol which included an initial pre-enrichment in BPW for 6–7 h at 37 ± 1.0°C.

**Figure 1:**
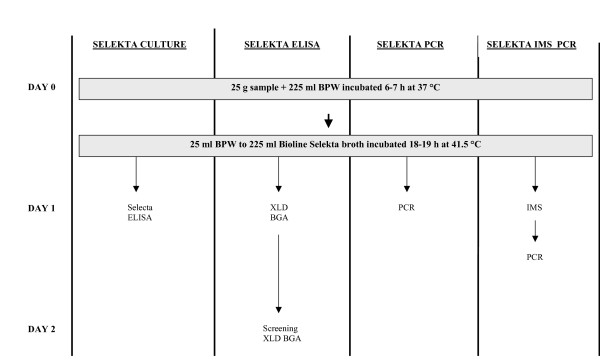
Flowchart of salmonella detection methods starting with the pre-enrichment step of BPW for 6–7 h (The Selekta enrichment protocol).

A flow chart of all methods that started with this pre-enrichment set is presented in Figure 1.

**Figure 2:**
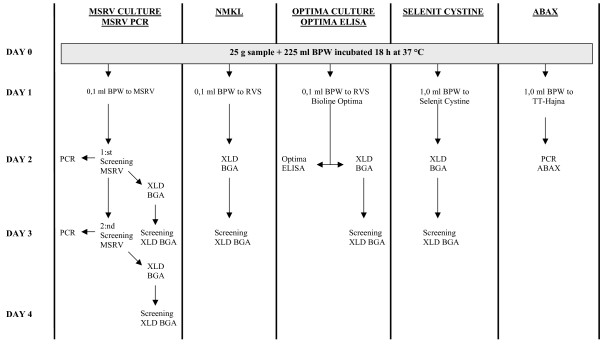
Flowchart of salmonella detection methods starting with the pre-enrichment step of BPW for 18–19 h.

The second pre-enrichment was started at 3 pm using BPW at ambient temperature. This set was used for all the other methods that were based on an initial pre-enrichment in EE BPW for 18 ± 1 h at 37 ± 1.0°C. A flow chart of all methods that started with this pre-enrichment set is presented in Figure 2.

### NMKL method (no.71, version 5)

Salmonella analysis according to NMKL (Nordic Committee on Food Analysis) 71:5: 1999. Pre-enrichment (with BPW) for 18 ± 1 h at 37° ± 1.0°C whereafter 0.1 ml BPW was added to 10 ml Rappaport-Vassiliadis Broth (RVS)(CM 866; Oxoid). RVS tubes were incubated for 18–24 h at 41.5 ± 0.5°C. One loop 10 μl of RVS was inoculated to Xylose-Lysin-Desoxycholate agar (XLD)(Lab M; Axel Johnson Lab System Inc. Solna, Sweden) supplemented with 1.5% Novobiocin. One loopful was also inoculated to Brilliant -Green-Phenol-Red agar (BGA)(Oxoid). The agar plates were incubated at 37°C h before screening for suspected salmonella colonies.

A preliminary positive or a definite negative answer is obtained by this method, at the earliest 3 days after the analyses have been initiated. Preliminary positive samples must be further confirmed by biochemical testing and serological agglutination.

### Selenite/Cystine method

The Selenite/Cystine method was only evaluated in Trials 7–9 with artificially contaminated cattle faecal samples.

Pre- enrichment (with BPW) for 18 ± 1 h at 37° ± 1.0°C, whereafter 1.0 ml of BPW was added to 10 ml Selenite/Cystine broth (In house, according to ISO 6579:1993 3. ed, National Veterinary Institute). Selenite/Cystine tubes were incubated for 18–24 h at 37 ± 1.0°C. One 10 μl loop of broth was inoculated to XLD agar (Lab M) (supplemented with 1.5 % Novobiocin) and BGA agar (Oxoid) respectively. The agar plates were incubated for 18–24 h at 37 ± 1.0°C before screening for suspected salmonella colonies.

A preliminary positive or definite negative is obtained with this method at the earliest 3 days after the analyses have been initiated. Preliminary positive samples must be further confirmed by biochemical testing and serological agglutination

### MSRV method

The MSRV method was performed according to Draft Amendment ISO 6579:2002/amendedDAmd 1 (2006-09-12) Amendment 1 Annex D: Detection of Salmonella spp. in animal faeces and in samples from the primary production stage, which is suggested as a new addendum to ISO 6579.

Pre-enrichment (with BPW) for 18 ± 1 h at 37 ± 1.0°C whereafter Modified Semi-solid Rappaport Vassiliadis agar plates (MSRV)(Oxoid CM 0910), supplemented with 1.0% Novobiocin were inoculated with three drops (a total of 0.1 ml) of BPW. MSRV agar plates were incubated for 18–24 h at 41.5 ± 0.5°C whereafter they were screened for suspected growth of salmonella. Agar plates were subsequently incubated for a further 18–24 h followed by a second screening for suspected salmonella growth. If suspected growth of salmonella was detected plating out was performed to both XLD agar (Lab M) (supplemented with 1.5% Novobiocin) and BGA (Oxoid). The plates were incubated for 18–24 h at 37° ± 1.0°C before screening for suspected salmonella colonies.

With this method a preliminary positive answer is obtained 3 days after the analyses have been initiated. A definite negative answer is obtained at the earliest after 4 days. Preliminary positive samples on XLD and BGA agar plates must be further confirmed by biochemical testing and serological agglutination.

### Vidas SLM (ELISA)

The enrichment protocol used in our study was as recommended by the manufacturer at the time the study was performed. (Since conclusions of the study, the manufacturer has altered the enrichment recommendations for faeces samples).

Pre-enrichment (with BPW) for 18 ± 1 h at 37 ± 1.0°C whereafter after 0.1 ml of BPW was added to 10 ml RVS(CM 866; Oxoid). RVS tubes were incubated for 7 h at 41.5 ± 0,2°C whereafter 0.5 ml of RVS was transferred to 10 ml of M-broth (supplied by the manufacturer). M broth tubes were incubated for 18–24 h at 41.5 ± 0.5°C. The Vidas SLM (ELISA) was then performed according to the manufacturer's instructions.

SLM with this enrichment protocol gives at the earliest definite negative answers or preliminary positive results 2 days after the analyses have been initiated.

Positive preliminary results must be confirmed by subjecting the RVS tubes to further incubation (simultaneous with inoculation to M broth) for up to 18–24 h at 41.5 ± 0.5°C. If Vidas SLM indicates a positive result, plating out is performed to XLD and BGA from RVS. This confirmation step for VidasSLM is equivalent to the above described NMKL method.

### Bioline Optima (ELISA)

Pre-enrichment (with BPW) for 18 ± 1 h at 37 ± 1.0°C, whereafter 0.1 ml of BPW was added to 10 ml RVS (supplied by the manufacturer). RVS tubes were incubated for 18–24 h at 41.5 ± 0.2°C. The Bioline Optima ELISA test was performed according to the manufacturer's instructions.

The Bioline Optima method gives at the earliest definite negative or preliminary positive results 2 days after the analyses have been initiated. Positive preliminary results must be confirmed by plating out from the RVS tubes to XLD and BGA agar plates, see below.

### Bioline Optima culturing method

The method was performed as described above. 10 μl RVS broth (RVS supplied by the manufacturer) was plated to XLD agar (Lab M) (supplemented with 1.5% Novobiocin) and BGA (Oxoid) respectively. Agar plates were incubated at for 18–24 h 37 ± 1.0°C before screening for suspected salmonella colonies

A preliminary positive or a definite negative answer is at the earliest obtained after 3 days after the analyses have been initiated. Preliminary positive samples must be further confirmed by biochemical testing and serological agglutination

This method is the confirmation procedure for Bioline Optima (ELISA). The method with RVS broth as a second enrichment step is equivalent to the NMKL method; see above.

### Bioline Selecta (ELISA)

Bioline Selecta was performed according to the manufacturer's instructions.

Pre-enrichment was initiated with BPW pre-warmed to 37°C and pre-enrichment was performed for 6–7 h at 37 ± 1.0°C. (The manufacturer's instruction is pre-enrichment in pre-warmed BPW for 6–10 h.) After pre-enrichment, 20 ml of BPW were transferred to 200 ml Selecta broth pre-warmed to 41.5°C. (Selecta broth supplied by the manufacturer). Selecta enrichment was performed in stomacher-bags for 18–19 h at 41.5 ± 0.5 C°. (The manufacturer's instruction is enrichment in pre-warmed SELECTA broth for 18–24 h.) After Selecta enrichment the Selecta ELISA was performed according to the manufacturer's instructions.

The Bioline Selecta's ELISA-test gives at the earliest definite negative answers or preliminary positive results 1 day after the analyses have been initiated. Positive preliminary results must be confirmed by plating out from the Selecta enrichment broth to XLD and BGA agar plates, which is the confirmation procedure for Bioline Selekta; see below.

### Bioline Selecta culturing method

Whilst performing the Bioline Selecta (ELISA), the Selecta broth was inoculated to selective agar plates, by plating 10 μl broth to both XLD agar (Lab M) and BGA (Oxoid). The results obtained were also the confirmation procedure for Bioline Selecta (ELISA).

A preliminary positive or definite negative answer is at the earliest obtained after 2 days, at the earliest, preliminary positive samples must be further confirmed by biochemical testing and serological agglutination.

### Automated BAX, BAX^® ^salmonella detection kit

BAX^® ^salmonella endpoint PCR analysis was performed according to the manufacturer's instructions, using a DNA processing protocol suggested for faecal samples where problems with inhibition of the PCR reaction are suspected.

Pre-enrichment (with BPW) for 18 ± 1 h at 37° ± 1.0°C whereafter 1.0 ml BPW was added to 10 ml TT-Hajna (Difco). TT-Hajna tubes were incubated for 18–24 h at 41.5 ± 0.5°C. Five microliter of the enriched sample was transferred into 200 microliter lysis buffer, as provided by the manufacturer. The samples were incubated at 37°C for 20 min, followed by 95°C for 10 min. These DNA preparations were saved frozen at -25°C. After for 3 months the samples were thawn, and mixed with a "PCR supplement solution" provided by the manufacturer, whereafter the endpoint PCR was performed in the A-BAX^® ^cycler/detector PCR machine, using a pre-programmed cycling protocol.

A preliminary positive result or a definite negative result is at the earliest obtained 2 days after the analyses are initiated. At the time of the study, the protocol used for BAX^® ^was a draft protocol, as suggested by the manufacturer, and the protocol we received did not include any specified confirmation step. Therefore, no confirmation step for the BAX^® ^method was used.

### The PCR method used for all in-house PCR based methods

PCR was performed according to Hoorfar et al. [17]. To prevent contamination, the preparation of reaction mixtures, DNA extraction, amplification and detection of PCR products were all performed in different laboratories. Aerosol-resistant filter pipette tips were used throughout the experiment.

Amplification of the salmonella *invA *gene was carried out in a 50-μl reaction mixture containing a 900-nM concentration of each primer (SalF: TCGTCATTCCATTACCTACC, SalR: AAACGTTGAAAAACTGAGGA) (Thermo Electron Corporation, Waltham, MA, USA), 100 nM salmonella probe (6-FAM- d(TCTGGTTGATTTCCTGATCGCA)-BHQ-1) (Thermo Electron Corporation), 100 nM internal control probe (mdC-ROX-d(CAACCAATGATGCCCGTTCCT)-BHQ-2) (Thermo Electron Corporation) 2.5 U of rTth DNA polymerase with Buffer Packs (Applied Biosystems, Foster City, Cal. USA), 5 μl of 10× chelating buffer (Applied Biosystems), 4 mM MgCl2, 0.2 mM each of deoxynucleoside triphosphates (Applied Biosystems), 8% (vol/vol) glycerol (molecular biology grade; Sigma, St. Louis, MO, USA), and double-distilled water to 50 μl. Standard template volume was 10 μl. The reaction mixture was subjected to amplification by using an automated DNA thermal cycler Rotorgene 2000 or 3000, in a real-time PCR format (Corbett Research; Mortlake, Australia). The amplification reaction was started by a heating step at 95°C for 6 min. A protocol of 45 cycles followed, where each cycle involved heating to 95°C for 30 s, and cooling to 55°C for 1 min. Negative controls with the DNA template substituted to water in the reaction mixture were included in each PCR run, as well as negative and positive DNA control samples, prepared from *E. coli *(ATCC 35218) and *S*. Typhimurium (CCUG 31969) obtained from the Culture Collection, University of Göteborg (CCUG), Sweden. If needed, amplicons were visualized on 2.0% agarose gels with 100 base-pair ladder as molecular weight markers (Amersham Biosciences, Little Chalfont, Bucks, England).

### In-house PCR – MSRV followed by real-time PCR analysis

The MSRV method was performed as described above. In parallel, when plating selective agar plates from suspected salmonella growth from the MSRV agar plates a 1 μl loop was dipped into the zone of growth on the MSRV-agar plate and then transferred to PCR tubes containing the master mix prepared as described above. A negative answer or preliminary positive PCR result is at the earliest obtained 3 h after the suspected growth zones on MSRV agar plates have been detected.

### In-house PCR – Bioline Selecta enrichment followed by real-time PCR analysis

Using the incubated Selecta enrichment broth, of which an aliquot had been saved at -20°C until analysis was performed, a two-step dilution series of 1:10 dilutions was performed in TE-buffer [pH 7.4]. 10 μl of diluted Selecta broth was added to the PCR tubes containing the master mix prepared as described above, where after real-time PCR analysis was performed.

A preliminary positive and definite negative result is obtained after one day. Confirmation of the preliminary positive results is done by the Selecta culturing protocol; see above.

### In-house PCR – Bioline Selecta followed by immuno-magnetic separation, DNA extraction and real-time PCR analysis

Immuno-magnetic separation (IMS), using anti salmonella antibodies, was performed on the overnight Selecta broth according to the manufacturers instructions (Dynal). The retrieved magnetic beads were stored frozen at -25°C for 2 months, after which they were thawed and transferred to a DNA extraction robot (BioRobot M48; Qiagene GmbH, Hilden, Germany). DNA was extracted with the MagAttract^® ^DNA Mini M48 kit (Qiagene). The eluate was used as template for real-time PCR as described above.

A preliminary positive and definite negative result with this protocol is at the earliest obtained after day one. Confirmation of the preliminary positive results is done by applying the Selecta culturing protocol (see above).

### Confirmation of suspected salmonella from the different culture-based methods used in the study

For each culturing method where salmonella was detected by plating out and reading selective agar plates, XLD and BGA, the following procedure was used: suspected salmonella colonies, up to five colonies per XLD/BGA- agar plate, were re-cultivated on blue-agar plates. If a trial set including several spiking levels produced several XLD and BGA plates with suspected salmonella growth, the lowest spiking level, including at least six parallel samples, was used for confirmation. For samples seeded with salmonella, confirmation of a suspected colony included correct colony morphology and agglutination with the specific O antigen according to the particular serotype used. For control purpose, agglutination with NaCl was performed to exclude auto-agglutination.

For confirmation of all un-spiked samples with suspected growth, confirmation we used agglutination with polyvalent antiserum for Poly O and Poly H antigens, and biochemistry, including TSI, urea and lysine-decarboxylase.

For each trial set, one salmonella colony, isolated for confirmation, was also serotyped to verify that it was the strain used for seeding that was reisolated.

### Extended attempts for isolation of salmonella from the assumed salmonella-free sample that proved positive by the BAX^® ^salmonella detection methods but negative by all other methods

**Figure 3:**
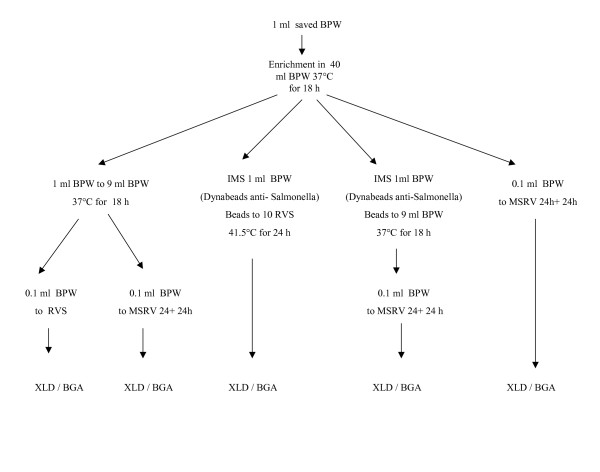
Extended attempts for isolation of salmonella from the assumed salmonella-free sample that proved positive by the BAX^® ^salmonella detection methods but negative by all other methods.

From each set of experiments, a 1 ml aliquot of the pre-enriched BPW was kept at -25°C. In order to attempt to isolate salmonella from the sample suspected of being salmonella-positive (by BAX^®^), this sample was used. The sample was thawed rapidly at 50°C to preserve as many viable bacteria as possible. Extensive attempts were performed to isolate salmonella from the saved BPW sample. The different isolation procedures are described in Figure 3.

### Statistical analysis

The statistical analysis was in accordance with the principles used in a method evaluation done by the European Commission Directorate General Joint Research Centre: Determination of Processed Animal Protein (PAPS) including meat and bone meal (MBM in feed. Parts I and II).

We believe that only samples to which we added salmonella were actually salmonella positive. All faecal samples used in the study were obtained from randomly selected Swedish farms, and were considered by us to be salmonella-negative. Our assumption is supported by the fact that none of the culture-based methods used in the study could detect salmonella in any of the un-seeded samples analysed.

Depending on these assumptions the results of the analyses can be categorized in these terms, which also are used in Tables 1, 2, 3, 4, 5.

TP True Positives

The analysis correctly detected salmonella in a sample when salmonella had been added

TN True Negatives

The analysis correctly failed to detected salmonella in a sample when salmonella had not been added

FP False Positives

The analysis had incorrectly detected salmonella in a sample when salmonella had not been added

FN False Negatives

The analysis had incorrectly not detected salmonella in a sample where salmonella had been added

The results from the different methods have been evaluated in Tables 1, 2, 3, 4, 5, using these terms

Accuracy (AC)=TP+TNTP+TN+FP+FN
 MathType@MTEF@5@5@+=feaafiart1ev1aaatCvAUfKttLearuWrP9MDH5MBPbIqV92AaeXatLxBI9gBaebbnrfifHhDYfgasaacH8akY=wiFfYdH8Gipec8Eeeu0xXdbba9frFj0=OqFfea0dXdd9vqai=hGuQ8kuc9pgc9s8qqaq=dirpe0xb9q8qiLsFr0=vr0=vr0dc8meaabaqaciaacaGaaeqabaqabeGadaaakeaacqqGbbqqcqqGJbWycqqGJbWycqqG1bqDcqqGYbGCcqqGHbqycqqGJbWycqqG5bqEcqqGGaaicqGGOaakcqqGbbqqcqqGdbWqcqGGPaqkcqGH9aqpdaWcaaqaaiabbsfaujabbcfaqjabgUcaRiabbsfaujabb6eaobqaaiabbsfaujabbcfaqjabgUcaRiabbsfaujabb6eaojabgUcaRiabbAeagjabbcfaqjabgUcaRiabbAeagjabb6eaobaaaaa@4E27@

Accuracy (AC) is a measure of the ability of a method to correctly classify samples containing salmonella as salmonella-positive, and samples not containing salmonella as salmonella- negative.

Sensitivity (SE)=TPTP+FN
 MathType@MTEF@5@5@+=feaafiart1ev1aaatCvAUfKttLearuWrP9MDH5MBPbIqV92AaeXatLxBI9gBaebbnrfifHhDYfgasaacH8akY=wiFfYdH8Gipec8Eeeu0xXdbba9frFj0=OqFfea0dXdd9vqai=hGuQ8kuc9pgc9s8qqaq=dirpe0xb9q8qiLsFr0=vr0=vr0dc8meaabaqaciaacaGaaeqabaqabeGadaaakeaacqqGtbWucqqGLbqzcqqGUbGBcqqGZbWCcqqGPbqAcqqG0baDcqqGPbqAcqqG2bGDcqqGPbqAcqqG0baDcqqG5bqEcqqGGaaicqGGOaakcqqGtbWucqqGfbqrcqGGPaqkcqGH9aqpdaWcaaqaaiabbsfaujabbcfaqbqaaiabbsfaujabbcfaqjabgUcaRiabbAeagjabb6eaobaaaaa@4960@

Sensitivity (SE) shows a method's ability to classify a sample containing salmonella as positive for salmonella.

Specificity (SP)=TNTN+FP
 MathType@MTEF@5@5@+=feaafiart1ev1aaatCvAUfKttLearuWrP9MDH5MBPbIqV92AaeXatLxBI9gBaebbnrfifHhDYfgasaacH8akY=wiFfYdH8Gipec8Eeeu0xXdbba9frFj0=OqFfea0dXdd9vqai=hGuQ8kuc9pgc9s8qqaq=dirpe0xb9q8qiLsFr0=vr0=vr0dc8meaabaqaciaacaGaaeqabaqabeGadaaakeaacqqGtbWucqqGWbaCcqqGLbqzcqqGJbWycqqGPbqAcqqGMbGzcqqGPbqAcqqGJbWycqqGPbqAcqqG0baDcqqG5bqEcqqGGaaicqGGOaakcqqGtbWucqqGqbaucqGGPaqkcqGH9aqpdaWcaaqaaiabbsfaujabb6eaobqaaiabbsfaujabb6eaojabgUcaRiabbAeagjabbcfaqbaaaaa@4914@

Specificity (SP) shows how reliably a method can classify a negative sample as being negative.

## Competing interests

The author(s) declares that there are no competing interests.

## Authors' contributions

EE outlined the design and coordinated the study, performed the statistical analysis and drafted the manuscript. AA coordinated and evaluated the molecular analysis and helped to draft the manuscript. Both authors read and approved the final manuscript
